# Towards the integration and development of a cross-European research network and infrastructure: the DEterminants of DIet and Physical ACtivity (DEDIPAC) Knowledge Hub

**DOI:** 10.1186/s12966-014-0143-7

**Published:** 2014-11-22

**Authors:** Jeroen Lakerveld, Hidde P van der Ploeg, Willemieke Kroeze, Wolfgang Ahrens, Oliver Allais, Lene Frost Andersen, Greet Cardon, Laura Capranica, Sebastien Chastin, Alan Donnelly, Ulf Ekelund, Paul Finglas, Marion Flechtner-Mors, Antje Hebestreit, Ingrid Hendriksen, Thomas Kubiak, Massimo Lanza, Anne Loyen, Ciaran MacDonncha, Mario Mazzocchi, Pablo Monsivais, Marie Murphy, Ute Nöthlings, Donal J O’Gorman, Britta Renner, Gun Roos, Abertine J Schuit, Matthias Schulze, Jürgen Steinacker, Karien Stronks, Dorothee Volkert, Pieter van’t Veer, Nanna Lien, Ilse De Bourdeaudhuij, Johannes Brug, on behalf of the DEDIPAC consortium

**Affiliations:** EMGO Institute for Health and Care Research, VU University Medical Center, van der Boechorststraat 7, 1081 BT Amsterdam, The Netherlands; Leibniz Institute for Prevention Research and Epidemiology -BIPS, Bremen, Germany; INRA, UR1303 ALISS, F-94205 Ivry-sur-Seine, France; University of Oslo, Oslo, Norway; Faculty of Medicine and Health Sciences, Ghent University, Ghent, Belgium; University of Rome Foro Italico, Rome, Italy; Glasgow Caledonian University, School of Health and Life Science, Scotland, UK; Centre for Physical Activity and Health Research, University of Limerick, Limerick, Ireland; Department of Sport Medicine, Norwegian School of Sport Sciences, Oslo, Norway; Institute of Food Research, Norwich, UK; Division of Sports and Rehabilitation Medicine, University of Ulm, Ulm, Germany; Netherlands Organisation for Applied Scientific Research (TNO), Leiden, The Netherlands; Johannes Gutenberg University, Mainz, Germany; Department of Neurological and Movement Sciences, University of Verona, Verona, Italy; Department of Statistical Sciences of the University of Bologna, Bologna, Italy; Department of Public Health and Primary Care, Institute of Public Health, University of Cambridge, Cambridge, UK; Sport & Exercise Sciences Research Institute, University of Ulster, Newtownabbey, UK; Department of Nutrition and Food Science, Rheinische Friedrich-Wilhelms-Universität Bonn, Bonn, Germany; Centre for Preventive Medicine, School of Health and Human Performance, Dublin City University, Dublin, Ireland; Department of Psychology, University of Konstanz, Constance, Germany; National Institute for Consumer Research, Oslo, Norway; Centre for Nutrition, Prevention and Health Services, National Institute for Public Health and the Environment, Bilthoven, The Netherlands; German Institute of Human Nutrition Potsdam-Rehbruecke, Potsdam, Germany; Department of Public Health, Academic Medical Centre, University of Amsterdam, Amsterdam, The Netherlands; Institute for Biomedicine of Aging, Friedrich-Alexander-Universität Erlangen-Nürnberg, Erlangen, Germany; Division of Human Nutrition, Wageningen University, Wageningen, The Netherlands; Department of Nutrition, University of Oslo, Oslo, Norway

**Keywords:** Diet, Physical activity, Sedentary behaviour, Joint programming, Lifestyle, Prevention, Measurement, Determinants, Interventions, Policy

## Abstract

To address major societal challenges and enhance cooperation in research across Europe, the European Commission has initiated and facilitated ‘joint programming’. Joint programming is a process by which Member States engage in defining, developing and implementing a common strategic research agenda, based on a shared vision of how to address major societal challenges that no Member State is capable of resolving independently. Setting up a Joint Programming Initiative (JPI) should also contribute to avoiding unnecessary overlap and repetition of research, and enable and enhance the development and use of standardised research methods, procedures and data management. The Determinants of Diet and Physical Activity (DEDIPAC) Knowledge Hub (KH) is the first act of the European JPI ‘A Healthy Diet for a Healthy Life’. The objective of DEDIPAC is to contribute to improving understanding of the determinants of dietary, physical activity and sedentary behaviours. DEDIPAC KH is a multi-disciplinary consortium of 46 consortia and organisations supported by joint programming grants from 12 countries across Europe. The work is divided into three thematic areas: (I) assessment and harmonisation of methods for future research, surveillance and monitoring, and for evaluation of interventions and policies; (II) determinants of dietary, physical activity and sedentary behaviours across the life course and in vulnerable groups; and (III) evaluation and benchmarking of public health and policy interventions aimed at improving dietary, physical activity and sedentary behaviours. In the first three years, DEDIPAC KH will organise, develop, share and harmonise expertise, methods, measures, data and other infrastructure. This should further European research and improve the broad multi-disciplinary approach needed to study the interactions between multilevel determinants in influencing dietary, physical activity and sedentary behaviours. Insights will be translated into more effective interventions and policies for the promotion of healthier behaviours and more effective monitoring and evaluation of the impacts of such interventions.

**Electronic supplementary material:**

The online version of this article (doi:10.1186/s12966-014-0143-7) contains supplementary material, which is available to authorized users.

## Background

Health is a key driver of Europe’s growth and prosperity and European governments are struggling with the growing social and economic consequences of an alarming increase in lifestyle related diseases, including obesity, cardiovascular disease, diabetes and cancer [1]. A healthy diet, sufficient physical activity and the avoidance of an overly sedentary lifestyle are key determinants of health across the life course –from preconception and in utero growth [2] to healthy ageing [3]. Improving health by providing citizens with the motivation, ability and opportunities to maintain a healthy lifestyle is a priority for most EU Member States in an effort to reduce the risk of lifestyle-related diseases. In order to facilitate this, a more focused and integrated approach using a common methodology is needed. This approach is required to investigate what the main risk- and health-promoting behaviours are, how these behaviours change over time and what the main determinants of exposure to and engagement in these health behaviours are. In addition, the approach is necessary to understand how policy and multilevel interventions affect these determinants and behaviours in an effort to promote health across Europe and how these policy interventions can be monitored and evaluated most effectively.

To address major societal challenges and enhance cooperation in research across Europe, the European Commission has initiated and facilitated ‘joint programming’. Joint programming is a process by which Member States engage in defining, developing and implementing a common strategic research agenda, based on a shared vision of how to address societal challenges that no Member State is capable of resolving independently.

Such joint programming across European countries can help to pool resources to enable more comprehensive and larger-scale research with more variation in exposures and outcomes, avoid unnecessary overlap and repetition and enable and enhance the development and use of standardised research methods, procedures, tools, infrastructure and data management necessary for high-quality research. It should thus contribute to improving and aligning the research infrastructure, including human capital, so that this can be used more effectively and efficiently. In addition, joint programming not only involves aligning or defining joint research agendas, but also combining and/or pooling research funding from the participating countries.

Within the EU Committee for Scientific and Technical Research, the High-Level Group on Joint Programming has identified and substantiated various themes for Joint Programming Initiatives (JPIs), one of which is ‘A Healthy Diet for a Healthy Life (HDHL)’. The JPI HDHL was then adopted by twelve Member States that set up a management and development structure and produced a strategic research agenda [4]. In this agenda, the JPI vision is formulated as such: ‘by 2030 all Europeans will have the motivation, ability and opportunity to consume a healthy diet from a variety of foods and have healthy levels of physical activity, and that the incidence of lifestyle-related diseases will have decreased significantly.’

The first act of this JPI is to realise joint programming, collaboration and harmonisation to further research on the determinants of diet and physical activity. Therefore the Determinants of Diet and Physical Activity (DEDIPAC) Knowledge Hub (KH) was started to establish a European transdisciplinary research network programme on determinants of diet and physical activity and their relationship to best-practice implementation strategies for long-term behaviour changes.

In this paper, we share this new European initiative in the field of behavioural nutrition and physical activity by describing the focus, organisation, aims, management and dissemination of the DEDIPAC KH and discussing the expected pros and cons and the challenges of European Joint Programming in this field.

### The focus of DEDIPAC

The overarching goal for DEDIPAC is to understand and be able to enact the most effective ways of improving public health through interventions targeting motivation, ability and opportunity to adopt and maintain healthy dietary, physical activity and sedentary behaviours [4]. In order to realise more effective promotion of healthy diets and physical activity across Europe, it is necessary to enhance and harmonise the measurement and monitoring of dietary, physical activity and sedentary behaviours. In addition, their individual, socio-cultural and environmental determinants must be integrated in order to contribute to planned population health promotion [5].

The DEDIPAC KH aims to contribute to building the research network and infrastructure and perform preparatory work to be able to better face this challenge. More specifically, the DEDIPAC KH aims to contribute to:

1. Enabling a better standardised and more continuous pan-European ‘needs analysis’ , i.e. to monitor dietary, physical activity and sedentary behaviours and changes in these behaviours across the life course and within populations to identify targets and target populations for (policy) interventions;
2. Exploring the main correlates and determinants of these behaviours in and across populations to help to tailor policies and interventions to target these determinants;
3. Learning from successes and failures of previous and on-going interventions and policies in order to improve evaluation and increase effectiveness of future interventions and policies and to identify and benchmark best practices across Europe and compare these internationally.

### Structure

The DEDIPAC KH comprises three Thematic Areas (TAs) that will be strongly interlinked and will work in parallel to cover the logical path from assessment and surveillance to public health interventions and policies via the exploration and modelling of determinants of dietary, physical activity and sedentary behaviours. The three TAs are characterised as follows:

- TA 1: Assessment and harmonisation of methods for future research, surveillance and monitoring and evaluation of interventions and policies;
- TA 2: Determinants of dietary, physical activity and sedentary behaviours across the life course and in vulnerable groups;
- TA 3: Evaluation and benchmarking of public health interventions and policies aimed at improving dietary, physical activity and sedentary behaviours across the life course.

Each of the TAs consist of different Work Packages (WPs) that constitute specific tasks that will be conducted by collaborating experts from different research groups within the DEDIPAC KH. Each of the TAs will deliver harmonised or aligned infrastructures, methodologies and/or evidence that will be compiled in a ‘toolbox’ available to scientists, health promotion professionals and policy makers. This toolbox will be made accessible via the DEDIPAC website (www.dedipac.eu). The three TAs and their backgrounds are described in more detail below.

### Thematic area 1: ‘Assessment and harmonisation’

To effectively develop and evaluate behavioural nutrition and physical activity interventions and policies that can be compared, translated, benchmarked and/or applied across different countries in Europe, the variability in methods to assess and monitor dietary, physical activity and sedentary behaviours and their determinants needs to be addressed first. At present, these are often study- and/or country specific and are not harmonised. Harmonised and more standardised measurement and monitoring are important prerequisites to compare evidence from different countries and to enable valid cross-country determinant research and policy evaluation. Moreover, existing national and emerging European infrastructures for surveillance, determinant research and interventions must be evaluated to assess the potential for further development of pan-European research infrastructures that can harbour these harmonised instruments.

The overall objective of this TA is to provide the pan-European research community with a harmonised set of reliable and valid measurement methods to be used for future research on dietary, physical activity and sedentary behaviours and their individual, socio-cultural and environmental determinants.

**Figure 1:**
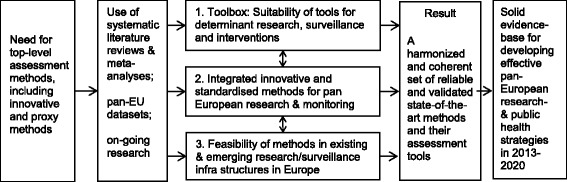
**Logic framework for the tasks in Thematic Area 1 ‘Assessment and harmonisation of methods for future research, surveillance and monitoring and evaluation of interventions and policies’.**

A harmonised and coherent set of reliable and valid state-of-the-art methods and assessment tools such as this will be identified by means of an evidence-based and expert-led overview of current and emerging assessment methodologies. As shown in Figure 1, this inventory of ‘state-of-the-art’ methods and tools will include:

1. Evaluation of their suitability for research on determinants, monitoring and surveillance of dietary, physical activity and sedentary behaviours and for evaluation of public health interventions and policies. The evaluated methods and tools will make up the contents of the DEDIPAC toolbox, which will be made freely available to the whole research community.
2. Identification of gaps in current methods/tools as well as the needs and requirements for future pan-European applications of such methods. This will strengthen the foundation for developing new integrated assessment tools, by combining the previously separate assessment methods of dietary, physical activity and sedentary behaviours. The identification of research and knowledge gaps will be based on the ‘prioritised topics‘described earlier, as well as on the inventory of topics covered in previous research.
3. The feasibility and potential integration of these newly developed tools in on-going European surveillance and research infrastructures will be evaluated and aligned with emerging pan-European research and surveillance infrastructures.

Dietary assessment methods and their limitations have been described in detail in several publications (e.g., [6,7]). The most widely-used, traditional methods of assessing total dietary intake are food frequency questionnaires (FFQ), 24-hour recalls and food records/diaries. These are all self-report methods and are subject to considerable measurement error and bias. Biomarkers providing objective information on diet could be an alternative, but these are only available for some selected nutrients and foods. Most evaluation studies of dietary assessment methods have assessed validity and reliability of food and nutrient intake. Much less is known about methods for assessing meal patterns and new and innovative methods under development such as web-based tools, mobile phone-based tools and camera tools. There have also been limited attempts to collect systematic information about methods that could be used across countries and cultures in Europe. Simpler, shorter and more cost-effective methods for measuring specific foods or dietary behaviours of interest in future pan-European monitoring, determinant and intervention research are needed. Moreover, the integration of novel technology needs to be considered since it has the potential to make these methods more time- and cost-effective [8].

Physical activity and sedentary behaviours have traditionally been assessed by self-reporting in large-scale cohort studies and surveillance systems. However, objective assessment methods, such as accelerometry, heart rate monitoring and combined accelerometry and heart rate monitoring have emerged as potential methods to assess physical activity and sedentary behaviours in large scale studies as well [9,10]. While much research has been conducted on the feasibility, validity and reliability of these methods to assess physical activity, less is known about these measurement techniques when used to assess sedentary behaviour. Therefore, the best available method of assessing both physical activity and sedentary behaviours in large-scale surveillance systems need to be identified.

Systematic literature reviews will help to identify relevant reliability and validation studies on assessment of dietary, physical activity and sedentary behaviours and their determinants. First, reference instruments will be identified (preferably (alloyed) gold standards) for dietary behaviour, physical activity and sedentary behaviours. Then the performance of existing proxy measurements will be assessed in terms of validity and reliability and both consistency and sources of heterogeneity of the assessment methods will also be identified. Prototyping and piloting innovative assessment methods will be initiated by mapping databases of on-going research. In addition, existing pan-European datasets on reference methods (e.g. independent replications of 24-hour recalls for diet or objective activity monitors for physical activity and sedentary behaviours) will be exploited to identify the main contributors to relevant variation (between countries, risk groups, age, sex, meals) in dietary, physical activity and sedentary behaviours. This will result in the description of the European diversity and at the same time stimulate and elicit potential innovative short-cut measurement methods to be explored in a feasibility pilot study. The range of relevant dietary, physical activity and sedentary behaviours and their determinants is very broad and priorities must be set. To efficiently select the most relevant contents, we will make an inventory of topics covered in earlier research and prioritise specific areas for further research in the DEDIPAC consortium. Prioritisation of topics will assist in maintaining focus and relevance in terms of public health challenges in specific stages of the life course. For example, but without anticipating any decisions, diet related topics could include sugar-sweetened beverage or fruit and vegetable intake in schoolchildren, or vitamin D and cobalamin supplementation in community-dwelling and institutionalised older people. Similar considerations will shape the selection of topics for physical activity and sedentary behaviour.

### Thematic area 2: ‘Determinants of dietary, physical activity and sedentary behaviour’

Dietary, physical activity and sedentary behaviours are determined by interactions between biological, psychological, sociological, economic, ecological, socio-economic and cultural factors, which are at least partly modifiable [11-13]. Although there is a wealth of knowledge on the individual impact of most of these factors, there is limited knowledge about the impact of their interplay and interactions on behavioural nutrition, physical activity and sedentariness, how they change over the life course, and how the determinants and their interactions differ between socio-cultural, and environmental contexts.

**Figure 2:**
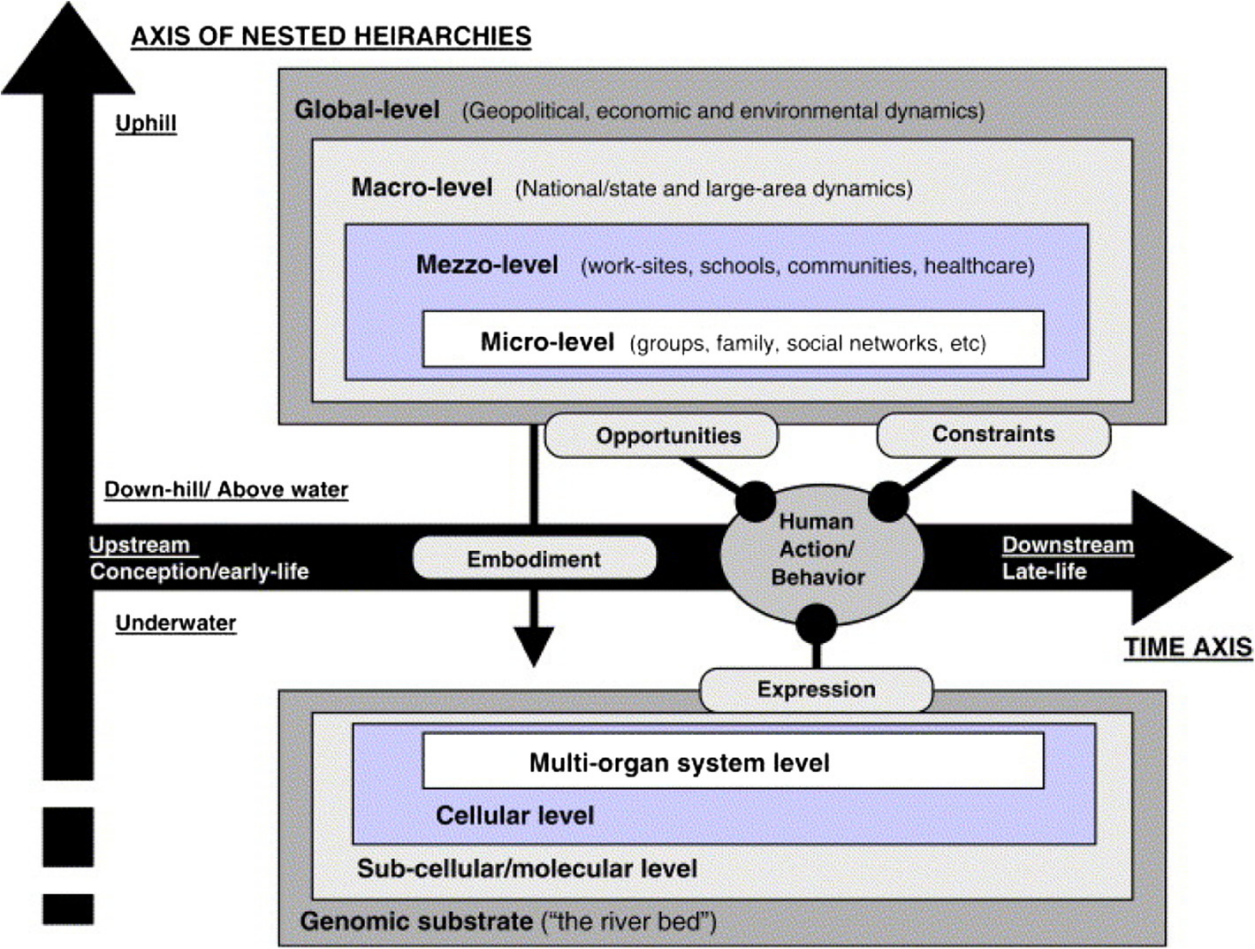
**Example of a determinant framework incorporating biological and social-ecological perspectives across the life course** [14]**.**

Several frameworks and models describe the theoretical relationship between the different levels and categories of determinants of behavioural nutrition and physical activity. For example, Glass and McAtee [14], have merged perspectives from the natural and social sciences by adding the biological system to a social-ecological model in which the different levels of influence (micro, meso, macro, global) indicate the proximity of the determinants to the individual engaging in the behaviour. This model incorporates the life course perspective by acknowledging potentially different determinants at different life stages, as well as the accumulative impact of behaviour on health and quality of life [14] (Figure 2). This model can be further enriched by the ANGELO-framework [15] to differentiate between the types of environmental influences and contexts that could be at play at each level (socio-cultural, physical, economic and political). Another potential addition to such models in DEDIPAC’s transdisciplinary approach could be the consideration of feedback loops and endogeneity effects explored in the economics literature (e.g. [16]), in which behaviours also affect the potential determinants. The Glass & McAtee ‘starting model’ provides a number of entry points for a linkage between biological (e.g. genetic) determinants/factors and more upstream/distal determinants. Specific actions related to this are being taken within the scope of DEDIPAC, including the GIS mapping of neighbourhood environments of twin pairs, which, when examining behaviour-environment associations, would allow a form of ‘control’ for genetic predisposition, age, sex, cohort effects, most maternal influences and other known (and unknown) factors. In short, Glass and McAtee take into consideration the different determinants and levels in a systems approach, extending beyond a linear line of influence. Their model –combined with insights from other models– will provide the basis for the further exploration of determinants within DEDIPAC.

Initially, we will explore, apply and enrich this framework for the three separate behaviours or behavioural categories and will progressively try to integrate insights across the three behaviours to generate knowledge on the interplay between common determinants of these behaviours. Cross-country comparisons and exploration of these multilevel determinants in pooled (secondary) data analyses will contribute to gaining further insight in this regard. This focus on combining and integrating behavioural nutrition and physical activity research closely matches with the focus of the International Society of Behavioural Nutrition and Physical Activity.

This TA will develop a European multi-disciplinary network and framework for determinant research in Europe by means of a two-stage process conducted in each of the work packages, each focusing on one of the behaviours, i.e. diet, physical activity and sedentary behaviour:

1. With the Glass & McAtee model and ANGELO framework as points of departure, review and integrate the current state-of-the art determinant frameworks (uni- or multi-disciplinary).
2. Proof-of-concept explorative secondary data analysis using state-of-the-art statistical methods (conducted within European transdisciplinary research networks) and explorative case studies of social inequalities and ethnic minorities.

A mix of established methods will be used, including systematic literature reviews and scoping reviews, a multi-disciplinary Delphi consensual process and expert workshops (to which individuals from a variety of disciplines will be invited, which will do justice to the multi-facetted conceptual framework depicted in Figure 2), as well as secondary analyses of cross-European data. For the latter, we will be using analytical techniques such as multilevel mediation and moderation analysis, structural equation modelling of pooled data, meta-analyses of different studies, and others. In this first phase of DEDIPAC, we will not be collecting primary data.

This second TA in DEDIPAC thus aims to understand the determinants of dietary, physical activity and sedentary behaviours, at both the individual and group levels, using a broad multi-disciplinary approach. In service of this multi-disciplinary and inter-behavioural scope, the use of a variety of data sources from different disciplines, focusing on different behaviours, determinants and aggregation levels is essential. There are already many disciplines involved in the Knowledge Hub – although not all relevant disciplines are fully covered yet. The disciplines that are represented currently include, among others: food and nutritional science (from molecular, epidemiological, anthropologic, dietic and public health perspectives), sport and physical activity sciences, (from epidemiological, exercise, sports medicine and biomedical perspectives), psychology (from general to health-specific, social and cognitive), biology, geography, public health, (health) economy, general/clinical medicine, informatics, engineering for mobile devices, statistics, medical biometry, sociology, policy, natural sciences, biomedicine, metabolic physiology, genomics, human ecology, environmental science, mathematics, Agro-Food Marketing etc.

However, this multidisciplinary approach will generate a major challenge in achieving harmonisation and matching across data sources as well as data sharing across research groups. Novel ways to perform pooled analysis of individual-level data without actually sharing the data will be explored. For instance, EU BioSHaRE established a password-protected web portal that facilitated remote and federated analyses of the harmonised datasets conducted at each participating institution using DataSHIELD software [17]. Another example of data sharing and harmonisation is the International Children’s Accelerometer Database (ICAD) that pools youth accelerometer data and reanalyses raw data files in order to harmonize data between different studies [18]. ICAD is closely linked to DEDIPAC and will serve as an example for other harmonisation exercises within DEDIPAC. Further examination of these and other approaches to data harmonisation and sharing is one of the research infrastructure issue areas in which DEDIPAC aims to make a difference, paving the way for European collaboration and JPI’s in the years to come.

### Thematic area 3: ‘Multilevel intervention, policy evaluation and benchmarking’

As stated in the JPI-HDHL strategic research agenda, DEDIPAC’s ultimate goal is to contribute to improving public health through interventions, actions and policies targeting dietary and physical activity behaviours [4]. TA 3 will use the information derived from the first two TAs to translate it into recommendations for health-promotion interventions and policies and to draft a DEDIPAC- toolbox for use in future policy and intervention monitoring, evaluation and benchmarking. TA 3 will focus on health promotion activities that are linked to the systems-oriented and social-ecological model of determinants (as described under TA 2) i.e. multilevel or multi-component interventions and actions that have the potential to reach large segments of the population, such as regional or national public health policies.

*Multilevel or multi-component interventions* are defined as theory-based interventions that use knowledge of the behavioural determinants at different levels (i.e. individual, socio-cultural and environmental) to improve dietary, physical activity and sedentary behaviours in individuals. We will focus in particular on multi-component interventions that take a social-ecological approach because literature reviews suggest these multi-component interventions are potentially the most effective [19-21]. These multi-component interventions are often not yet translated into policies to improve the target behaviours, but they could be translated into policies if adopted by governmental agencies in the future. They can therefore be regarded as feasibility/pilot interventions to inform future policy making.

Several frameworks for evaluating both multi-component interventions and public policies aimed at enhancing physical activity [22] or diet [23] already exist in several countries. DEDIPAC will make use of the methods and experience of the International Network for Food and Obesity/NCDs Research, Monitoring and Action Support (INFORMAS [24]) when developing the toolbox for TA 3. INFORMAS was recently founded to monitor and benchmark food environments and policies globally. Standardised protocols and indicators are being developed to measure and benchmark the extent of government policy implementation and private sector actions and practices [24]. In addition, for four key modules (food prices, provision, promotion and retail), ‘environmental equity’ indicators are being developed to assess progress towards reducing diet-related health inequalities. The impacts of national policies are very difficult to measure (they are rarely amenable to randomised controlled trials) and an analysis of a rich data series measuring levels of policy implementation, impacts on food environments and health outcomes is one of the few robust ways of evaluating national policies. It is foreseen that the integration of these methodologies, measures and tools into a dynamic and evolving toolbox will contribute to better understanding of the complexity of multiple interactions by developing new approaches and combining data on dietary behaviours and also on physical activity and sedentary behaviours with individual, social and environmental factors. The scientific concept underpinning this TA draws heavily on implementation science, which focuses on closing the gap between evidence on interventions and their translation into effective policies and programmes [25].

TA 3 will utilise the DEDIPAC- toolbox developed in TA 1 and the individual, socio-cultural- and environmental-level determinants of dietary, physical activity and sedentary behaviours described by TA 2 to develop an evaluation framework and open-access database in which policy and multi-component interventions will be described. This will include their evaluation in terms of reach, effectiveness, adoption, implementation and maintenance. This pan-European framework will then be pilot tested in ongoing interventions or policies in a number of DEDIPAC Member States and will be adapted accordingly. Eventually, the framework will outline evidence- and practice-based policy and multi-component intervention options.

In order to fulfil the aforementioned aims, the draft evaluation framework will first be pilot-tested by 19 partners across nine countries using ‘natural experiments’ or policies that are currently being implemented or have recently been implemented. An expert consensus meeting with a variety of stakeholders will be organised to agree upon a final framework. Getting their input is an essential step in shaping the end-product. A final symposium will also be organised and held to inform and consult stakeholders –with separate and joint sessions for scientists, policy makers, health promotion professionals- about the results, conclusions, recommendations and continuation of the DEDIPAC Knowledge Hub.

### Management and dissemination

A consortium of 46 consortia, research institutes and groups from 12 EU Member States has been established (please refer to Additional file 1 for a full list of scientists and Member States involved). As is the custom in these large, multi-site, international programmes, the work in the three TAs has been translated into specific tasks and deliverables in eleven WPs. The work is to be completed in three years and coordinated by a management team led by the KH coordinator. The progress and further development of this JPI is monitored and advised upon by a scientific advisory board as well as a stakeholder advisory board.

The progress and deliverables will be communicated and disseminated via the DEDIPAC website (www.dedipac.eu), scientific publications and non-scientific publications in English and other languages, consortium meetings, as well as in a series of workshops specifically aimed at early-career researchers. The workshops will address the conduct of systematic literature reviews, secondary data analysis and/or advanced statistical methods, for instance.

## Discussion

The DEDIPAC KH, as described in the current paper, is the first act of the European JPI HDHL. It is a serious attempt to join forces across countries in Europe to align research focus, infrastructure and funding. It will also enable the better use of the rich European research community and infrastructure and avoid redundancy in the field of behavioural nutrition and physical activity. It is a first experiment to test whether or not joint programming such as this is indeed possible and meaningful and adds value in addition to –or instead of— national research and European Commission research programming and funding. The results generated will be of importance to policy-makers, researchers, professionals in the area of public health, the food industry and citizens. DEDIPAC KH’s focus is on developing the most effective ways of improving public health through interventions for motivating and enabling consumers to adopt and maintain healthy diets and initiate and maintain physical activity. As such, this JPI has adopted a focus very close to the mission of the International Society for Behavioural Nutrition and Physical Activity.

The large variability in diet, physical activity and sedentary behaviour patterns observed across Europe, together with a great deal of diversity in political, economic, socio-cultural, physical environmental and policy contexts as well as differences in the prevalence of health conditions, provides a unique scientific opportunity to learn more about the determinants of dietary, physical activity and sedentary behaviours [26-29]. Europe is – in principle – a great living research laboratory: there is much variety in terms of determinants and behaviour, policies and interventions. However, the research arena in this field is somewhat scattered across Europe because each country sets its own research priorities, has its own funding schemes, uses different methods and measures to assess and monitor the same or very similar behaviours and determinants and sometimes even publishes in a different language. Joint programming and the establishment of a network of researchers and research infrastructure with a common purpose –directly endorsed and (financially) supported by the ministries of the participating countries— has great potential. It can contribute significantly to the necessary harmonisation, enabling valid cross-European comparisons and thus learning from progress, pitfalls and mistakes made in other countries or regions and avoiding redundancy in terms of research.

One could argue that joint programming and building a network such as DEDIPAC is not needed or necessary because there are European Commission research programmes that already exist, i.e. the framework programmes and now Horizon2020. These research programmes are, by definition, coordinated and programmed at a cross-European level and adding JPI actions such as DEDIPAC may only further complicate the pan-European research infrastructure, organisation and funding arenas. However, European Commission funding constitutes only a minor part of total investment in behavioural nutrition and physical activity research across Europe [4]. The recent negotiations on and the adoption of the multi-annual financial framework for the European Union for 2014–2020 shows that, at present, European Member States are not willing or able to substantially increase spending on European Commission-funded research. In addition to the European Commission-funded research programmes, European Union (EU) Member States have their own research programmes and funding schemes. This approach includes research on healthy eating and physical activity and, to date, these funding schemes lack true coordination, despite the fact that research priorities in the European countries in this field are very similar or in fact overlap. Additionally, Europe lags behind the United States and Japan in the percentage of gross domestic product invested in research and development. This combination of lower, less coordinated and more scattered research spending will set Europe back in the field of behavioural nutrition and physical activity research. Better alignment and coordination of research funds between Member States can thus make a significant difference. DEDIPAC is a first experiment to see if this can indeed work in practice.

Another question is whether joint programming is suitable for behavioural nutrition and physical activity research. Quite a large number of European Commission-funded studies have been or are being conducted to gain more insight into determinants of diet and physical activity in different age groups across Europe. Previous and currently-running European projects such as HOPE, EURO-PREVOB, ENERGY, HELENA, IDEFICS, I-FAMILY, SPOTLIGHT, ALPHA and others have focused on determinants of dietary, physical activity and sedentary behaviours, made inventories of and recommended policies. However, even in these projects, different measurement instruments are often used and no larger-scale attempt has yet been made to harmonise and integrate the data and/or results of these projects. The institutional base of DEDIPAC is much larger than the aforementioned EU-funded projects. In addition, its multidisciplinarity and its scope is also broader. It addresses three big themes simultaneously (aligned in three thematic areas), covering three different lifestyle behaviours. DEDIPAC will make use of, but will also go beyond what has been done is separate EU funded projects, aiming to work towards agreed upon measurement standards for cross European research.

A major strength of the DEDIPAC KH is its ability to facilitate the simultaneous study of dietary, physical activity and sedentary behaviours, their determinants and the policies and multi-component interventions that target these determinants and behaviours. Although these behaviours comprise sets of different specific behaviours influenced by a range of personal, socio-cultural and environmental determinants, they form the two sides of the energy-balance equation and thus need to be targeted simultaneously or at least in relation to each other in order to promote synergy and avoid negative compensation. The DEDIPAC KH will help to identify common ground between dietary, physical activity and sedentary behaviours with regard to assessment, surveillance, determinants and intervention strategies. Ultimately, this will facilitate better synchronisation in diet and physical activity research, policy and practice.

As a potential downside, it should be acknowledged that working in multi-disciplinary teams from a multitude of research institutes across 12 countries (at present, but other countries have expressed interest in joining DEDIPAC), with 8 different main languages, with financial support from as well as reporting to 12 different funding agencies, is already a major organisational endeavour, to say the least. In the context of a complex organizational structure such as this, it is even more challenging to reach consensus about harmonisation of measures, benchmarking of policies, data sharing and other big issues. Nevertheless, the DEDIPAC consortium was able to write, submit and receive approval for the DEDIPAC proposal from the 12 countries, all in a matter of about four months’ time. Furthermore, all WPs have started and are working according to schedule in the first several months after the official start of DEDIPAC, i.e. 1 December 2013.

The current DEDIPAC consortium has good coverage of the different relevant disciplines. Because DEDIPAC originates in the JPI HDHL, experts in human nutrition are very well represented. The focus of DEDIPAC was extended to also include physical activity and sedentary behaviour at an early stage and these areas of expertise are therefore also very much involved. However, DEDIPAC’s focus on multilevel determinants towards a more systems-level approach to behavioural determinants and including policy evaluation and benchmarking, requires involvement of a range of social and behavioural expertise that needs further strengthening within the consortium. From the very start and made explicit in the approved proposal, DEDIPAC will be open to other groups and will, wherever necessary, actively try to include new groups with expertise to further enrich the KH. This first DEDIPAC act will help to expand on existing collaborations and assist in building new ones while identifying expertise gaps within the KH. This may help the KH to stay relevant in the longer term. Another way to improve the long-term sustainability of the DEDIPAC approach and community are the dissemination and capacity building activities. These activities aim to expand the DEDIPAC community and to train early-career scientist in this multi-disciplinary approach.

## Conclusion

The DEDIPAC KH uses joint research programming and funding across different countries in Europe to work towards better research harmonisation and collaboration in the field of behavioural nutrition and physical activity research. As a first joint action in this joint programming initiative, DEDIPAC has already made an excellent start in setting up a complex, cross-country organisational structure.

## Additional file

Additional file 1:
**Full consortium of the DEDIPAC Knowledge Hub.**

## Abbreviations

DEDIPAC: Determinants of diet and physical activity
HDHL: A healthy diet for a healthy life
JPI: Joint programming initiative
KH: Knowledge hub
TA: Thematic area
WP: Work package
