# Supplementary material for: Towards the integration and development of a cross-European research network and infrastructure: the DEterminants of DIet and Physical ACtivity (DEDIPAC) Knowledge Hub
Source: Int J Behav Nutr Phys Act. 2014 Nov 22;11:143. doi: 10.1186/s12966-014-0143-7 (PMC4245771; doi:10.1186/s12966-014-0143-7)
Supplement: Additional file 1: — Full consortium of the DEDIPAC Knowledge Hub. [file 12966_2014_143_MOESM1_ESM.docx]

**Additional file 1**

**Full consortium of the DEDIPAC Knowledge Hub** (on alphabetical order of country and surname)

| **Austria** |  |
| --- | --- |
| P. Hofmann | Medical University Graz |
| M. Mayrhofer | Medical University Graz |
| S. Wallner | Medical University Graz |
| K. Zatloukal | Medical University Graz |
|  |  |
| **Belgium** |  |
| G. Cardon | Ghent University |
| P. Clarys | Vrije Universiteit Brussel |
| K. de Cocker | Ghent University |
| S. de Henauw | Ghent University |
| I. Debourdeaudhuij | Ghent University |
| B. Deforche | Ghent University/Vrije Universiteit Brussel |
| L. Maes | Ghent University |
| I. Sioen | Ghent University |
| D. van Dyck | Ghent University |
|  |  |
| **Finland** |  |
| R. Korpela | University of Helsinki |
| H. Mykkanen | University of Eastern Finland, Department of Clinical Nutrition |
| S. Virtanen | National Institute for Health and Welfare |
|  |  |
| **France** |  |
| O. Allais | L'Institut National de la Recherche Agronomique (INRA) |
| S. Blanc | Institut Pluridisciplinaire Hubert Curien |
| S. Blanchemanche | AgroParisTech |
| Y. Boiri | L'Institut National de la Recherche Agronomique (INRA) |
| P. Dargent | Centre de Recherche en Nutrition Humaine Rhône-Alpes |
| P. Even | L'Institut National de la Recherche Agronomique (INRA) |
| C. Feart | Institut National de la Santé et de la Recherche Médicale (INSERM) |
| C. Gaudichon | AgroParisTech |
| C. Guillet | L'Institut National de la Recherche Agronomique (INRA) |
| S. Issanchou | L'Institut National de la Recherche Agronomique (INRA) |
| D. Langin | Centre de Recherche en Nutrition Humaine Rhône-Alpes |
| M. Laville | Centre de Recherche en Nutrition Humaine Rhône-Alpes |
| E. Lefai | Centre de Recherche en Nutrition Humaine Rhône-Alpes |
| J.-A. Nazare | Centre Européen Nutrition Santé |
| J.-M. Oppert | University Pierre et Marie Curie-Paris |
| V. Requillart | Toulouse School of Economics |
| J. Saussede | Centre de Recherche en Nutrition Humaine Rhône-Alpes |
| C. Simon | Centre de Recherche en Nutrition Humaine Rhône-Alpes |
| N. Slimani | International Agency for Research on Cancer |
| C. Sulmont | L'Institut National de la Recherche Agronomique (INRA) |
| A. Vuillemin | Université de Lorraine, APEMAC |
| C. Weber | Centre de Recherche en Nutrition Humaine Rhône-Alpes |
|  |  |
| **Germany** |  |
| W. Ahrens | Leibniz Institute for Prevention Research and Epidemiology -BIPS, Bremen |
| H. Boeing | German Institute of Human Nutrition |
| T. Brand | Leibniz Institute for Prevention Research and Epidemiology -BIPS, Bremen |
| F. Breyer | University of Konstanz |
| S. Brosig | Leibniz-Institute of Agricultural Development in Central and Eastern Europe |
| V. Bruschi | Leibniz-Institute of Agricultural Development in Central and Eastern Europe |
| A. Bub | Max Rubner-Institut |
| C. Buck | Leibniz Institute for Prevention Research and Epidemiology -BIPS, Bremen |
| C. Burggraf | Leibniz-Institute of Agricultural Development in Central and Eastern Europe |
| I. Dolgopolova | Leibniz-Institute of Agricultural Development in Central and Eastern Europe |
| U. Ebner-Priemer | institüt für Sport und Sportwissenschaft |
| R. Ensenauer | Ludwig-Maximilians-Universität München |
| M. Flechtner-Mors | University Ulm |
| E. Freiberger | Friedrich-Alexander-Universität Erlangen-Nürnberg (FAU) |
| H. Hauner | Else Kroener-Fresenius-Center for Nutritional Medicine |
| A. Hebestreit | Leibniz Institute for Prevention Research and Epidemiology -BIPS, Bremen |
| T. Heuer | Max Rubner-Institut |
| D. Hipp | institüt für Sport und Sportwissenschaft |
| G. Hirschfelder | Regensburg University |
| I. Hoffman | Max Rubner-Institut |
| S. Hoffmann | Christian-Albrechts-Universität Kiel |
| I. Hoffmann | Max Rubner-Institut |
| S. Hummel | Institute of Diabetes Research, Helmholtz Zentrum München |
| D. Kahlert | Stuttgart University |
| T. Kubiak | Johannes Gutenberg University Mainz |
| J. Linseisen | Helmholtz Zentrum München |
| W. Meyerhof | German Institute of Human Nutrition Potsdam-Rehbruecke |
| K. Michels | University of Freiburg |
| R. Muche | Ulm University |
| M. Mueller-Albinsky | Leibniz-Institute of Agricultural Development in Central and Eastern Europe |
| U. Nöthlings | University of Bonn |
| M. Pflüger | Leibniz Institute for Prevention Research and Epidemiology -BIPS, Bremen |
| I. Pigeot | Leibniz Institute for Prevention Research and Epidemiology -BIPS, Bremen |
| T. Pischon | Max-Delbrück Center Berlin |
| A. Ploeger | Kassel University |
| O. Pollatos | Ulm University |
| B. Renner | University of Konstanz |
| H. Rohm | Technische Universität Dresden |
| W. Schlicht | Stuttgart University |
| H. Schulz | Helmholtz Zentrum München |
| M. Schulze | German Institute of Human Nutrition Potsdam-Rehbruecke |
| H. Schupp | University of Konstanz |
| U. Schwarz | Saarland University Saarbrücken |
| S. Sonnentag | University of Mannheim |
| J. Spranger | Charité-Universitätsmedizin Berlin |
| J. Steinacker | Ulm University |
| B. Thumann | Leibniz Institute for Prevention Research and Epidemiology -BIPS, Bremen |
| K. van Laerhoven | Technical University Darmstadt, Embedded Systems Lab |
| D. Volkert | Friedrich-Alexander-Universität Erlangen-Nürnberg (FAU) |
| R. von Kries | Ludwig-Maximilians-Universität München |
| P. Warschburger | University of Potsdam |
| A. Woll | Karlsruhe Institute of Technology |
| M. Wolters | Leibniz Institute for Prevention Research and Epidemiology -BIPS, Bremen |
| H. Zeeb | Leibniz Institute for Prevention Research and Epidemiology -BIPS, Bremen |
| A.G. Ziegler | The Institute of Diabetes Research (IDF), Helmholtz Zentrum München |
| B.C. Zyriax | University Hospital Hamburg-Eppendorf |
|  |  |
| **Ireland** |  |
| S.J. Belton | Dublin City University |
| C. Borham | University College Dublin |
| W. Brady | University College Cork |
| C. Burns | Cork Instiution of Technology |
| B. Carson | University of Limerick |
| F. Chambers | University College Cork |
| A. Clifford | University of Limerick |
| S. Coote | University of Limerick |
| T. Coppinger | Cork Institution of Technology |
| M. Coulter | St Patricks College of Education |
| A. Donnely | University of Limerick |
| J. Eustace | University College Cork |
| Y. Finn | National University of Ireland |
| A. Flynn | University College Cork |
| C. Foley-Nolan | Human Health & Nutrition, safefood |
| J. Harrington | University College Cork |
| C. Hayes | Trinity College Dublin |
| J. Issartel | Dublin City University |
| Ph. Jakeman | University of Limerick |
| C. Kelleher | University College Dublin |
| N. Kennedy | University of Limerick |
| A. Kirby | University College Cork |
| M. Lyons | University of Limerick |
| C. MacDonncha | University of Limerick |
| N. McCaffrey | Dublin City University |
| N. Murphy | Waterford Institution of Technology |
| C. Murrin | University College Dublin |
| E. Murtagh | Mary Immaculate College of Education |
| J. Nelson | University of Limerick |
| D. Ni Chroinin | Mary Immaculate College of Education |
| A. Nugent | University College Dublin |
| C. O’Gorman | University of Limerick |
| M. O’Sullivan | University of Limerick |
| D.J. O'Gorman | Dublin City University |
| G. O'Malley | Children's University Hospital |
| A. Parle-McDermott | Dublin City University |
| I. Perry | University College Cork |
| C. Sage | University College Cork |
| R. Sohun | University of Limerick |
| A. Staines | Dublin City University |
| M.R. Sweeney | Dublin City University |
| D. Tannehill | University of Limerick |
| G. Warrington | Dublin City University |
| C. Woods | Dublin City University |
| C. Woods | Dublin City University and University of Limerick |
|  |  |
| **Italy** |  |
| D. Berardi | Data Ananalysis Support Centre |
| M. Bianchi | Barilla |
| M. Biasiotti | Istituto di Teoria e Tecniche dell'Informazione Giuridica |
| G. Biolo | University of Trieste |
| S. Boccia | Catholic University of the Sacred Heart |
| L. Capranica | University of Rome Foro Italico |
| F. G. Di Girolamo | University of Trieste |
| L. Donini | Università di Roma La Sapienza |
| C. Fischer | Free University of Bozen |
| E. Ghigo | University of Turin |
| G. Napolitano | University of Chieti-Pescara |
| L. Iacoviello | Casa di Cura Montevergine |
| M. Lanza | University of Verona |
| G. Latorre | La Sapienza University, Rome |
| G. Lazzeri | University of Sienna |
| A. Lenzi | La Sapienza University, Rome |
| D. Lucini | University of Milan |
| M. Mazzocchi | University of Bologna |
| S. Migliaccio | University Foro Italico |
| M. A. Minetto | University of Turin |
| D. Moro | Università Cattolica del Sacro Cuore |
| A. Palma | University of Palermo |
| F. Pigozzi | University of Rome Foro Italico |
| N. Porro | Laboratorio di Ricerca Sociale |
| P. Portincasa | University of Bari |
| W. Ricciardi | Catholic University of the Sacred Heart |
| A. Saba | Agricultural Research Council – Centre of Food and Nutrition |
| F. Turchi | Istituto di Teoria e Tecniche dell'Informazione Giuridica |
| S. Zanuso | Technogym Study and Research Centre |
|  |  |
| **Norway** |  |
| L.F. Andersen | University of Oslo |
| S. A. Anderssen | Norwegian School of Sport Sciences |
| U. Ekelund | Norwegian School of Sport Sciences |
| N. Lien | University of Oslo |
| Y. Ommundsen | Norwegian School of Sport Sciences |
| G. Roos | National Institute for Consumer Research |
| G. Rugseth | Norwegian School of Sport Sciences |
| L. Terragni | Oslo and Akershus University College of Applied Sciences |
|  |  |
| **Poland** |  |
| J. Bajerska | Poznan University of Life Sciences |
| A. Bialek | Medical University of Silesia in Katowice |
| S. Bosiacki | Eugeniusz Piasecki University School of Physical Education |
| M. Bronikowska | Eugeniusz Piasecki University School of Physical Education |
| M. Bronikowski | University School of Physical Education |
| K. Horodyska | University of Social Sciences and Humanities |
| J. Jeszka | Poznan University of Life Sciences |
| Z. Kasprzak | Eugeniusz Piasecki University School of Physical Education |
| I. Laudanska | Akademia Wychowania Fizycznego |
| M. Laurentowska | Eugeniusz Piasecki University School of Physical Education |
| A. Luszczynska | University of Social Sciences and Humanities |
| J. Maciaszek | Eugeniusz Piasecki University School of Physical Education |
| A. Majchrzak | University of Pittsburgh |
| L. Pilaczynska | Eugeniusz Piasecki University School of Physical Education |
| B. Pluta | Eugeniusz Piasecki University School of Physical Education |
| R. Stemplewski | Eugeniusz Piasecki University School of Physical Education |
| M. Straczkowski | Polish Academy of Sciences |
| A. Suwalska | Poznan University of Medical Sciences |
| R. Szeklicki | Eugeniusz Piasecki University School of Physical Education |
| M. Tomczak | Akademia Wychowania Fizycznego |
| M. Wilski | Eugeniusz Piasecki University School of Physical Education |
| M. Wozniewicz | Poznan University of Life Sciences |
|  |  |
| **Spain** |  |
| I. Castano | CIBERDEM |
| R. Estruch | Centro de Investigación Biomédico en Red (CIBEROBN) |
| J.A.F. Formoso | Centro de Investigación Biomédico en Red (CIBEROBN) |
| E. Lurbe | University of Valencia and Centro de Investigación Biomédico en Red (CIBEROBN) |
| J.L. Peñalvo | Centro Nacional de Investigaciones Cardiovasculares |
| M. Sotos | Centro Nacional de Investigaciones Cardiovasculares |
| J. Vioque | Universidad Miguel Hernández de Elche |
|  |  |
| **the Netherlands** |  |
| S. Bausch | Netherlands Organisation for Applied Scientific Research |
| J. Brug | EMGO+ Institute for Health and Care Research |
| P. Dagnelie | Maastricht University |
| J. de Jong | Hanze University of Applied Sciences, Groningen |
| E. de Vet | Wageningen University and Research Centre |
| A. Geelen | Wageningen University and Research Centre |
| I. Hendriksen | Netherlands Organisation for Applied Scientific Research |
| J.S.M. Hobbelen | Hanze University of Applied Sciences, Groningen |
| H.A. Jager | Hanze University of Applied Sciences, Groningen |
| S. Kremers | Maastricht University |
| W. Kroeze | EMGO+ Institute for Health and Care Research |
| J. Lakerveld | EMGO+ Institute for Health and Care Research |
| A. Loyen | EMGO+ Institute for Health and Care Research |
| M. Nicolaou | Academic Medical Center/University of Amsterdam |
| G. Nijpels | EMGO+ Institute for Health and Care Research |
| C. Renders | Windesheim University of Applied Sciences |
| A. Ronteltap | Wageningen University and Research Centre |
| A. Roodenburg | HAS University of Applied Sciences |
| J. Schuit | Centre for Nutrition, Prevention and Health Services |
| H. Snoek | Wageningen University and Research Centre |
| I. Steenhuis | EMGO+ Institute for Health and Care Research |
| K. Stronks | Academic Medical Center/University of Amsterdam |
| M. van den Berg | Centre for Nutrition, Prevention and Health Services |
| H. van der Ploeg | EMGO+ Institute for Health and Care Research |
| C. van der Schans | Hanze University of Applied Sciences, Groningen |
| P. van 't Veer | Wageningen University and Research Centre |
| K. Zimmermann | Wageningen University and Research Centre |
|  |  |
| **United Kingdom** |  |
| J. Allan | University of Aberdeen |
| M.E. Barker | University of Sheffield |
| P. Bissell | University of Sheffield |
| C. Bleakley | University of Ulster |
| G. Breslin | University of Ulster |
| S. Chastin | Glasgow Caledonian University School of Health and Life Science |
| D. D. McGrath | University of Ulster |
| P. Dall | Glasgow Caledonian University School of Health and Life Science |
| G. W. Davison | University of Ulster |
| B. de Roos | University of Aberdeen |
| F. Douglas | University of Aberdeen |
| P. Finglas | Institute of Food Research |
| E. Goyder | University of Sheffield |
| L. Harvey | Institute of Food Research |
| M. Holdsworth | University of Sheffield |
| A. Ludbrook | University of Aberdeen |
| C. M. McClean | University of Ulster |
| G. McNeill | University of Aberdeen |
| A. McNeilly | University of Ulster |
| P. Monsivais | University of Cambridge |
| M. H. Murphy | University of Ulster |
| D. Skelton | Glasgow Caledonian University School of Health and Life Science |
| B. Stansfield | Glasgow Caledonian University School of Health and Life Science |
| E. Tulle | Glasgow Caledonian University School of Health and Life Science |
| E.A. Williams | University of Sheffield |
